# Modeling and Control Design of Port-Hamiltonian Systems in Discrete-Time †

**DOI:** 10.3390/e28091002

**Published:** 2026-09-07

**Authors:** Alessandro Macchelli

**Affiliations:** Department of Electrical, Electronic, and Information Engineering—“Guglielmo Marconi” (DEI), University of Bologna, viale del Risorgimento 2, 40136 Bologna, Italy; alessandro.macchelli@unibo.it; Tel.: +39-051-2093031

**Keywords:** discrete-time systems, port-Hamiltonian systems, modeling, control design, passivity

## Abstract

This paper aims to describe a synthesis procedure for discrete-time, energy-based regulators for continuous-time port-Hamiltonian systems. The methodology consists of three steps. The first deals with the definition of a discrete-time approximation of the plant, which is subsequently employed in the development of the control law. The discrete-time model is obtained from the continuous-time dynamics by replacing the gradient of the Hamiltonian function with a discrete gradient. In this way, passivity, with the energy as storage function, is preserved, although the resulting state equation is in implicit form. The second step concerns the control synthesis and extends the continuous-time energy-shaping plus damping injection design technique to the proposed class of discrete-time port-Hamiltonian systems. Finally, the last step addresses the interconnection between the digital controller and the continuous-time plant. The coupling is implemented via a zero-order hold and relies on the solution of an optimization problem that determines the “best” and “minimal” correction to be applied to the nominal control action in order to achieve the same performance as that obtained when the regulator is connected in closed loop with the discrete-time model of the plant. This is the reference scenario used to develop and tune the control law. The complete procedure (time discretisation, control design, and coupling implementation) is illustrated through an example.

## 1. Introduction

Port-Hamiltonian systems were introduced about thirty years ago to provide a unified framework for modeling lumped-parameter continuous-time physical systems [1]. Over the past decades, several control synthesis methodologies have been developed [2,3,4]. More recently, their discrete-time extension has become a popular framework for the geometric integration of ordinary differential equations (ODEs), paving the way for digital control system design. Time-integration schemes are designed to preserve either the “structure” or the “energy” of continuous-time systems, and the literature distinguishes between two main categories: symplectic and energy-preserving integrators. Discrete-time Hamiltonian and port-Hamiltonian systems based on symplectic integrators have been extensively employed, for example, in [5,6,7,8,9]. This work instead focuses on a class of port-Hamiltonian systems belonging to the family of energy-preserving integrators [10,11], with a particular emphasis on discrete-time control design.

The discrete-time port-Hamiltonian systems considered in this paper constitute a simple extension of those proposed in [12,13,14,15,16,17,18] and are characterized by dynamics that depend on the discrete gradient of the Hamiltonian [10] (Definition 3.1). Further details can be found in [19,20], while the extension to distributed parameter systems is presented in [21,22]. This particular dynamic structure is useful for control synthesis because it directly yields an energy balance relation for the discrete-time system, thereby simplifying both the design procedure and Lyapunov-based stability analysis. Its main drawback is that the state equation is implicit.

The control design procedure consists of three steps. The first is devoted to developing a discrete-time port-Hamiltonian approximation of the plant that belongs to the class illustrated before. Based on this discrete-time model, the second design step addresses the definition of the control action. One option is to rely on an extension of the energy-shaping plus damping-injection paradigm [2], for which several contributions have already been presented in the literature [9,12,19,20]. Here, we propose an extension of the Interconnection and Damping Assignment Passivity-based Control (IDA-PBC) framework [3]. Under the simplifying assumption that the interconnection and damping structures of the system are constant, the proposed approach extends the procedure presented in [20] (Section III.A). It is worth mentioning that notable contributions in this research area can also be found in [18,23,24]. The idea is to determine a state-feedback law capable of modifying the internal and dissipative structures of the open-loop system, as well as its Hamiltonian function. In particular, the “new” Hamiltonian is characterized by a (global) minimum at the desired configuration (energy shaping), while the dissipative structure is modified to introduce sufficient dissipation to make this minimum attractive (damping injection). As in continuous time, mapping the open-loop system into the desired closed-loop system requires solving a matching equation. In continuous time, this equation is a nonlinear partial differential equation (PDE), whereas in the digital case it is algebraic in the discrete gradient.

The feedback action influences the system by modifying both its energy function and its “structure”. These provide two effective degrees of freedom for shaping the steady-state and transient response. Since the regulator is designed and tuned in discrete time, the remaining task is to define a strategy for coupling the digital controller with the continuous-time plant. This is the third and final stage of the synthesis procedure. Within energy- and passivity-based approaches, the interfacing of digital and physical systems is commonly addressed by preserving passivity across the interconnection [25,26]. Here, we present the technique proposed in [27], which designs the interface to match the performance of the reference discrete-time scenario while introducing *minimal* modifications to the nominal control action. In this respect, a suitable performance measure is the variation in the closed-loop Hamiltonian (energy) function along the system trajectories. If this quantity decreases at every sampling instant by at least the same amount as in the discrete-time case, no performance loss occurs. These observations motivate an optimization problem whose objective function penalizes the magnitude of the perturbation applied to the nominal control action. Its constraints are defined by the continuous-time plant dynamics and an upper bound on the “desired” energy level at the end of each sampling interval. The proposed design procedure is illustrated using a two-degree-of-freedom planar manipulator.

The paper is organized as follows: In Section 2, a summary of discrete-time port-Hamiltonian systems is presented, while an overview of the energy-based control design and the IDA-PBC framework in discrete-time are discussed in Section 3. Section 4 contains the digital-to-analog interfaces arising from the solution of different optimization problems. The example is discussed in Section 5, where the complete design procedure is illustrated in detail. Conclusions are in Section 6.

## 2. Discrete-Time Port-Hamiltonian Systems

In this paper, we refer to the following class of continuous-time port-Hamiltonian systems Ref. [1]:

(1) $$\left\{\begin{array}{cc} \dot{x}\left(t\right) & =\left[J(x(t))-R(x(t))\right]\nabla H\left(x\left(t\right)\right)+G\left(x\left(t\right)\right)u\left(t\right)\\ y(t) & =G^{T}\left(x\left(t\right)\right)\nabla H\left(x\left(t\right)\right)\\ x(0) & =x_{0}. \end{array}\right.$$

In (1), $t\in R_{\geq 0}$ and $x\left(t\right)\in X\subseteq R^{n}$ are the time and state variable, u(t)∈U and y(t)∈Y, with $U,\;Y\subseteq R^{m}$, are the input and output, $J\left(x\right),\;R\left(x\right)\in R^{n\times n}$ are such that $J\left(x\right)=-J^{T}\left(x\right)$ and $R\left(x\right)=R^{T}\left(x\right)\geq 0$ for all x∈X, $G\left(x\right)\in R^{n\times m}$ is full-rank, H:X→R is the Hamiltonian (energy) function, and $x_{0}\in X$ the initial condition. The matrix-valued functions *J* and *R* represent the interconnection and the dissipative structures of the system, respectively. Along the system’s trajectories we have that

(2) $$\begin{array}{cc} \dot{H}\left(x\left(t\right)\right) & =-\nabla^{T}H\left(x\left(t\right)\right)R\left(x\left(t\right)\right)\nabla H\left(x\left(t\right)\right)+y^{T}\left(t\right)u\left(t\right)\\ \; & \leq y^{T}\left(t\right)u\left(t\right), \end{array}$$

so if *H* is lower-bounded, (1) is passive and *H* is the storage function.

The design of a digital control law for (1) relies on the discrete-time approximation proposed in [19,20]. In brief, such a discrete-time model is obtained at first by replacing the time derivative by the finite difference

(3) $$\dot{x}\left(t_{k}\right)≃\frac{1}{\tau }\left(x_{k+1}-x_{k}\right),$$

being $t_{k}=k\tau$ the time samples and $x_{k}=x\left(t_{k}\right)$, with τ>0 and k∈N. Secondly, a discrete approximation of the gradient operator is adopted; see e.g., Ref. [10] (Definition 3.1).

**Definition** **1.** *Let $\Phi :R^{p}\to R$ be a continuously differentiable function. A discrete gradient$\bar{\nabla }\Phi :R^{p}\times R^{p}\to R^{p}$ is a continuous map such that for all $\varphi ,\;\varphi_{+}\in R^{p}$ we have*

(4) $$\begin{array}{cc} {\left(\varphi_{+}-\varphi \right)}^{T}\bar{\nabla }\Phi \left(\varphi ,\varphi_{+}\right)\; & =\Phi \left(\varphi_{+}\right)-\Phi \left(\varphi \right), \end{array}$$

(5) $$\begin{array}{cc} \lim_{\varphi_{+}\to \varphi }\bar{\nabla }\Phi \left(\varphi ,\varphi_{+}\right)\; & =\nabla \Phi (\varphi ). \end{array}$$

Typical examples are the mean value discrete gradient [28] (Theorem 2.1) defined as

(6) $$\bar{\nabla }\Phi \left(\varphi ,\varphi_{+}\right)=\int_{0}^{1}\nabla \Phi \left(\left(1-\sigma \right)\varphi +\sigma \varphi_{+}\right)\;d\sigma ,$$

or the Gonzalez discrete gradient, Ref. [10] (Proposition 3.1). For a quadratic function $\Phi \left(\varphi \right)=\varphi^{T}Q\varphi$ with $Q=Q^{T}$, it is easy to check that $\bar{\nabla }\Phi \left(\varphi ,\varphi_{+}\right)=Q\left(\varphi +\varphi_{+}\right)$.

If we combine (1), (3) and Definition 1, for any k∈N, the discrete-time formulation of the port-Hamiltonian dynamics for control design is Refs. [19,20]:

(7) $$\left\{\begin{array}{cc} x_{k+1} & =x_{k}+\tau \left[\bar{J}\left(x_{k},x_{k+1}\right)-\bar{R}\left(x_{k},x_{k+1}\right)\right]\bar{\nabla }H\left(x_{k},x_{k+1}\right)+\tau \bar{G}\left(x_{k},x_{k+1}\right)u_{k}\\ y_{k} & ={\bar{G}}^{T}\left(x_{k},x_{k+1}\right)\bar{\nabla }H\left(x_{k},x_{k+1}\right), \end{array}\right.$$

with $u_{k}=u\left(t_{k}\right)$ and initial condition $x_{0}$. This class of discrete-time systems can be seen as an extension of [12,15,18]. The functions $\bar{J},\;\bar{R}:X\times X\to R^{n\times n}$ and $\bar{G}:X\times X\to R^{n\times m}$ are discrete approximations of *J*, *R* and *G*. More precisely, for all $x,\;x_{+}\in X$, we require that $\bar{J}\left(x,x_{+}\right)=-{\bar{J}}^{T}\left(x,x_{+}\right)$, $\bar{J}\left(x,x\right)=J\left(x\right)$, $\bar{R}\left(x,x_{+}\right)={\bar{R}}^{T}\left(x,x_{+}\right)\geq 0$, $\bar{R}\left(x,x\right)=R\left(x\right)$, and $\bar{G}\left(x,x\right)=G\left(x\right)$. Admissible choices for $\bar{J}$ are $J\left(\frac{1}{2}\left(x+x_{+}\right)\right)$, $\frac{1}{2}J\left(x\right)+\frac{1}{2}J\left(x_{+}\right)$ or simply J(x), and similarly for $\bar{R}$ and $\bar{G}$. From (4) and (7), we get the discrete-time version of (2):

(8) $$\begin{array}{cc} H\left(x_{k+1}\right)-H\left(x_{k}\right) & ={\bar{\nabla }}^{T}H\left(x_{k},x_{k+1}\right)\left(x_{k+1}-x_{k}\right)\\ \; & =-\tau {\bar{\nabla }}^{T}H\left(x_{k},x_{k+1}\right)\bar{R}\left(x_{k},x_{k+1}\right)\bar{\nabla }H\left(x_{k},x_{k+1}\right)+\tau y_{k}^{T}u_{k}\\ \; & \leq \tau y_{k}^{T}u_{k}. \end{array}$$

**Example** **1.** *In the linear case, the port-Hamiltonian system (1) takes the following form:*

(9) $$\left\{\begin{array}{cc} \dot{x}\left(t\right) & =\left(J-R\right)Qx\left(t\right)+Gu\left(t\right)\\ y(t) & =G^{T}Qx\left(t\right)\\ x(0) & =x_{0}, \end{array}\right.$$

*where $J,\;R\in R^{n\times n}$ are constant and such that $J=-J^{T}$ and $R=R^{T}\geq 0$, while $G\in R^{n\times m}$ is constant and full rank. In (9), the Hamiltonian function is quadratic, more precisely $H\left(x\right)=\frac{1}{2}x^{T}Qx$, with $Q=Q^{T}>0$, which implies that ∇H(x)=Qx. It is immediate to see that the discrete-time version of (9) is given by*

(10) $$\left\{\begin{array}{cc} x_{k+1} & =x_{k}+\frac{\tau }{2}\left(J-R\right)Q\left(x_{k}+x_{k+1}\right)+\tau Gu_{k}\\ y_{k} & =\frac{1}{2}G^{T}Q\left(x_{k}+x_{k+1}\right). \end{array}\right.$$

*Now, since the matrix $I-\frac{\tau }{2}\left(J-R\right)Q$ is invertible because the eigenvalues of the matrix (J−R)Q cannot have positive real part, the implicit state equation can be made explicit. Consequently, the state equation in (10) can be rewritten as*

$$x_{k+1}={\left[I-\frac{\tau }{2}\left(J-R\right)Q\right]}^{-1}\left[I+\frac{\tau }{2}\left(J-R\right)Q\right]x_{k}+\tau {\left[I-\frac{\tau }{2}\left(J-R\right)Q\right]}^{-1}Gu_{k}$$

*since $\bar{\nabla }H\left(x,x_{+}\right)=\frac{1}{2}Q\left(x+x_{+}\right)$, the explicit dynamics is described by a linear system with feedthrough.*

**Example** **2.** *Let us consider a mechanical system locally described by n configuration variables $q=\left(q_{1},\ldots ,q_{n}\right)\in R^{n}$. The kinetic energy is $\frac{1}{2}\dot{q}M\left(q\right)\dot{q}$, where $M\left(q\right)=M^{T}\left(q\right)>0$ for all q the generalized mass matrix, while we let V(q) to denote the potential energy. As illustrated in [1], if no constraints are present, the dynamics can be described by a port-Hamiltonian system in the form (1), more precisely:*

(11) $$\left\{\begin{array}{cc} \left(\begin{array}{c} \dot{q}\left(t\right)\\ \dot{p}\left(t\right) \end{array}\right)\; & =\left(\begin{array}{cc} 0 & I\\ -I & -D \end{array}\right)\left(\begin{array}{c} \frac{\partial H}{\partial q}\left(q\left(t\right),p\left(t\right)\right)\\ \frac{\partial H}{\partial p}\left(q\left(t\right),p\left(t\right)\right) \end{array}\right)+\left(\begin{array}{c} 0\\ B(q) \end{array}\right)u\left(t\right)\\ y(t) & =B^{T}\left(q\right)\frac{\partial H}{\partial p}\left(q\left(t\right),p\left(t\right)\right), \end{array}\right.$$

*where $p=M\left(q\right)\dot{q}$ are the generalized momenta, the Hamiltonian function is the total energy*

(12) $$H\left(q,p\right)=\frac{1}{2}p^{T}M^{-1}\left(q\right)p+V\left(q\right),$$

*$u,\;y\in R^{m}$ are the generalized forces and velocities, respectively. However, in (11), we assume that B(q) is full rank, while $D=D^{T}\geq 0$ takes into account the viscous friction forces, if present. It is easy to see that the discrete-time approximation of (11) is*

(13) $$\left\{\begin{array}{cc} q_{k+1} & =q_{k}+\tau {\bar{\nabla }}_{p}H\left(q_{k},p_{k},q_{k+1},p_{k+1}\right)\\ p_{k+1} & =p_{k}-\tau \left[{\bar{\nabla }}_{q}H\left(q_{k},p_{k},q_{k+1},p_{k+1}\right)-D{\bar{\nabla }}_{p}H\left(q_{k},p_{k},q_{k+1},p_{k+1}\right)\right]+\\ & \;\tau \bar{B}\left(q_{k},q_{k+1}\right)u_{k}\\ y_{k} & ={\bar{B}}^{T}\left(q_{k},q_{k+1}\right){\bar{\nabla }}_{p}H\left(q_{k},p_{k},q_{k+1},p_{k+1}\right), \end{array}\right.$$

*where $\bar{B}$ is a discrete approximation of B. For simplicity, we can set $\bar{B}\left(q_{k},q_{k+1}\right)=B\left(q_{k}\right)$. Since the Hamiltonian function in (12) is non-quadratic, it is not always possible to employ the mean value discrete gradient, as obtaining an explicit expression for the integral in (6) may be difficult or even impossible. In such cases, one may either resort to an approximation of the integral or adopt a different discrete gradient, such as the Gonzalez discrete gradient, which is constructed by sampling the continuous one. Finally, note that the state equation in (13) is given in implicit form. Consequently, unlike the linear case discussed in Example 1, an explicit state-update equation cannot, in general, be derived.*

**Remark** **1.** *System (7) is not based on a particular discrete gradient, which turns out to be a degree-of-freedom at disposal to the designer, together with the expression of $\bar{J}$, $\bar{R}$ and $\bar{G}$. Such a model for control design is a compromise between accuracy in the response and (numerical) complexity of the final control law. The drawback is that the state equation is implicit: this is the price to pay to have “for free” the energy balance relation (8), which is the starting point for control design and stability analysis.*

**Remark** **2.** *As mentioned in Remark 1 and reported in Examples 1 and 2, the state equation in (7) is in implicit form. It can be made explicit, i.e., solved for $x_{k+1}$ in the linear case discussed in Example 1 because H is quadratic and J, R and G in (1) are constants. This approach has been followed in [12,18]. In the nonlinear case, however, it is necessary to keep such an implicit formulation. Based on passivity arguments and inspired by [29], under mild conditions on the structure of the system and on the discrete gradient, in [20] it has been shown that trajectories exist independently from the sampling time. This is the same rationale pursued in [15] where a similar result has been proved under the hypothesis that the sampling time is sufficiently small. In this paper, we assume that the dynamical equations are well-posed, so the next state can always be (at least numerically) computed.*

## 3. Energy-Based Control Design in Discrete-Time

This section aims at generalizing the Interconnection and Damping Assignment Passivity-Based Control (IDA-PBC) design methodology [3], originally developed for continuous-time port-Hamiltonian systems, to the discrete-time setting. Remarkable contributions in this direction include [18,23,24], the latter being specifically focused on mechanical systems. These methodologies aim at assigning an equilibrium $x_{★}\in R^{n}$ to (7) by means of state feedback, while preserving the port-Hamiltonian structure. If the closed-loop system retains the port-Hamiltonian form, asymptotic convergence to $x_{★}$ can be established by relying on a balance relation similar to (8).

We start from the balance relation (8) for the open-loop dynamics (7), which can be compactly rewritten as

$$\tau^{-1}\left[H\left(x_{k+1}\right)-H\left(x_{k}\right)\right]=y_{k}^{T}u_{k}-d\left(x_{k},x_{k+1}\right),$$

where $d(x_{k},x_{k+1})\geq 0$ takes into account the dissipated power during the *k*-th sampling interval. Similarly to the continuous-time case [2,3], the idea behind the design of an energy-based state-feedback controller is to find

(14) $$u_{k}=\beta \left(x_{k},x_{k+1}\right)+v_{k}$$

so that the closed-loop dynamics obey the “new” balance relation

(15) $$\tau^{-1}\left[H_{d}\left(x_{k+1}\right)-H_{d}\left(x_{k}\right)\right]=z_{k}^{T}v_{k}-d_{d}\left(x_{k},x_{k+1}\right),$$

in which $H_{d}\left(x_{k}\right)$ is a “desired” energy with a (local) minimum at $x_{★}$, $d_{d}\left(x_{k},x_{k+1}\right)\geq 0$ replaces the natural dissipation, increased to improve the convergence rate, and $z_{k}$ is the new passive output.

For simplicity, we assume that the matrix-valued functions *J*, *R* and *G* in (1) are constant, and consequently that their discrete-time counterparts in (7) are also constant, i.e., $\bar{J}=J$, $\bar{R}=R$ and $\bar{G}=G$. The general case, in which these matrices depend on the state, is more involved. Nevertheless, most of the results and considerations developed for the simplified setting can be extended to the state-dependent case. The most natural extension of the IDA-PBC approach to the discrete-time setting requires to determine the feedback law $\beta (x_{k},x_{k+1})$ in (14) so that the state equation in (7) is mapped to

(16) $$x_{k+1}=x_{k}+\tau \left(J_{d}-R_{d}\right)\bar{\nabla }H_{d}\left(x_{k},x_{k+1}\right)+\tau Gv_{k},$$

which represents the “target” or “desired” dynamics. In (16), we have

(17) $$\begin{array}{cc} H_{d}\left(x_{k}\right) & =H\left(x_{k}\right)+H_{a}\left(x_{k}\right)\\ J_{d} & =J+J_{a},\;\mathrm{with}\;J_{a}=-J_{a}^{T}\\ R_{d} & =R+R_{a},\;\mathrm{with}\;R_{a}=R_{a}^{T}\geq 0, \end{array}$$

which includes the desired Hamiltonian function, interconnection matrix and dissipative structure, respectively. It is immediate to see that, if we let

(18) $$z_{k}=G^{T}\bar{\nabla }H_{d}\left(x_{k},x_{k+1}\right)=y_{k}+G^{T}\bar{\nabla }H_{a}\left(x_{k},x_{k+1}\right)$$

the target system (16) equipped with the output $z_{k}$ satisfies the balance relation (15).

The comparison between the open-loop dynamics (7) and the target one (16) shows that the control action $\beta (x_{k},x_{k+1})$ modifies the energy and the interconnection and dissipative structures of the original system. The rationale behind the choice of $H_{a}$, $J_{a}$ and $R_{a}$ is the same as in the continuous-time case; see, for example, [2,3] for more details. In brief, we can say that, as far as the so-called energy-shaping action is concerned, the function $H_{a}\left(x_{k}\right)$ is selected so that the closed-loop energy $H_{d}\left(x_{k}\right)$ in (17) has a minimum in $x_{★}$. However, $R_{a}$ is chosen to improve the convergence rate towards the minimum of the closed-loop energy function. A similar effect is achievable via the output feedback (damping injection) by imposing that $v_{k}=-\varphi \left(z_{k}\right)$ in (14), with the function φ such that $z^{T}\varphi \left(z\right)\geq 0$ for all $z\in R^{m}$, and $z_{k}$ defined in (18). The simplest damping injection law is

(19) $$v_{k}=-K_{z}z_{k}=-K_{z}G^{T}\bar{\nabla }H_{d}\left(x_{k},x_{k+1}\right)=-K_{z}\left[y_{k}+G^{T}\bar{\nabla }H_{a}\left(x_{k},x_{k+1}\right)\right],$$

with $K_{z}\in R^{m\times m}$ and $K_{z}=K_{z}^{T}\geq 0$. Note that such a control action can also be applied to the open-loop system (7) by setting $u_{k}=-\varphi \left(y_{k}\right)$. If $x_{★}$ is the only invariant solution of the steady state, the “new” dissipative structure in the system forces the asymptotic stability of the equilibrium. With simple calculations, we show that $H_{a}\left(x_{k}\right)$, $J_{a}$, $R_{a}$ and $\beta (x_{k},x_{k+1})$ have to satisfy the *matching equation:*

(20) $$G\beta \left(x_{k},x_{k+1}\right)=\left(J_{a}-R_{a}\right)\bar{\nabla }H\left(x_{k},x_{k+1}\right)+\left(J_{d}-R_{d}\right)\bar{\nabla }H_{a}\left(x_{k},x_{k+1}\right)$$

In the next proposition, sufficient conditions for the solution of (20) so that the feedback action (14) asymptotically stabilizes a forced equilibrium $x_{★}\in R^{n}$ of (7) are presented. This result is an immediate extension of [20] (Proposition 3.1).

**Proposition** **1.** *Under the assumption that the structure matrices in (7) are constant, denote by $x_{★}\in R^{n}$ a forced equilibrium, and let ${\bar{K}}_{a}:R^{n}\times R^{n}\to R^{n}$ be a function such that*

(21) $$G^{⊥}\;\left[\left(J_{a}-R_{a}\right)\bar{\nabla }H\left(x_{k},x_{k+1}\right)+\left(J_{d}-R_{d}\right){\bar{K}}_{a}\left(x_{k},x_{k+1}\right)\right]=0$$

*being $G^{⊥}\;$ the full-rank left annihilator of G, i.e., $G^{⊥}\;G=0$. If*

$$\frac{\partial K_{a}}{\partial x}\left(x\right)=\frac{\partial^{T}K_{a}}{\partial x}\left(x\right),$$

*with $K_{a}\left(x\right):={\bar{K}}_{a}\left(x,x\right)$, then there exists $H_{a}:R^{n}\to R$ for which $K_{a}\left(x\right)=\frac{\partial H_{a}}{\partial x}\left(x\right)$. However, if for all $x,\;\xi \in R^{n}$, we have*

$${\left(\xi -x\right)}^{T}{\bar{K}}_{a}\left(x,\xi \right)=H_{a}\left(\xi \right)-H_{a}\left(x\right);$$

*then, ${\bar{K}}_{a}$ is the discrete gradient of $H_{a}$, i.e., $\bar{\nabla }H_{a}\left(x,\xi \right)={\bar{K}}_{a}\left(x,\xi \right)$, and the control action (14) with*

(22) $$\begin{array}{cc} \beta (x_{k},x_{k+1}) & =G^{+}\left[\left(J_{a}-R_{a}\right)\bar{\nabla }H\left(x_{k},x_{k+1}\right)+\left(J_{d}-R_{d}\right){\bar{K}}_{a}\left(x_{k},x_{k+1}\right)\right]\\ \; & =G^{+}\left[\left(J_{a}-R_{a}\right)\bar{\nabla }H\left(x_{k},x_{k+1}\right)+\left(J_{d}-R_{d}\right)\bar{\nabla }H_{a}\left(x_{k},x_{k+1}\right)\right], \end{array}$$

*being $G^{+}$ the pseudo-inverse of G, maps (7) to the discrete-time system (16) with Hamiltonian function, interconnection and dissipative structures defined in (17) and output (18). Finally, if*

(23) $$\begin{array}{cc} \bar{\nabla }H\left(x_{★},x_{★}\right)+{\bar{K}}_{a}\left(x_{★},x_{★}\right)\; & =0\\ {\left(x-x_{★}\right)}^{T}\left[\bar{\nabla }H\left(x_{★},x\right)+{\bar{K}}_{a}\left(x_{★},x\right)\right] & \geq 0 \end{array}$$

*for all $x\in B_{\delta }\left(x_{★}\right)=\left\{\xi \in R^{n}:∥x-\xi ∥\leq \delta \right\}$ and for some δ>0, then $x_{★}$ is a locally stable equilibrium for the closed-loop system with $v_{k}=0$. Furthermore, if the largest invariant set contained in*

(24) $$\left\{x\in R^{n}\;|\;\exists \xi \in R^{n}\;s.t.\;{\bar{\nabla }}^{T}H_{d}\left(x,\xi \right)R_{d}\bar{\nabla }H_{d}\left(x,\xi \right)=0\right\}\cap B_{\delta }\left(x_{★}\right)$$

*is $\left\{x_{★}\right\}$, then $x_{★}$ is a locally asymptotically stable equilibrium for the closed-loop system with $v_{k}=0$.*

**Proof.**  The result can be proved in the same way as [20] (Proposition 3.1). Note that asymptotic stability is a consequence of the La Salle’s invariance principle. In fact, for the closed-loop system we have $H_{d}\left(x_{k+1}\right)-H_{d}\left(x_{k}\right)=-{\bar{\nabla }}^{T}H_{d}\left(x_{k},x_{k+1}\right)R_{d}\bar{\nabla }H_{d}\left(x_{k},x_{k+1}\right)\leq 0$, thus the corresponding energy function decreases until the trajectories evolve inside the set (24). Since the largest invariant set contained in (24) equals $\left\{x_{★}\right\}$, the result immediately follows. □

A crucial point in the methodology of Proposition 1 is the solvability of the matching Equation (21), which can be re-written in terms of the discrete gradient of $H_{a}$ as

(25) $$G^{⊥}\;\left[\left(J_{a}-R_{a}\right)\bar{\nabla }H\left(x_{k},x_{k+1}\right)+\left(J_{d}-R_{d}\right)\bar{\nabla }H_{a}\left(x_{k},x_{k+1}\right)\right]=0.$$

The simplest approach is to rely on the PDE that follows from (25) once $x_{k+1}\to x_{k}\equiv x$, i.e.,

(26) $$G^{⊥}\;\left[\left(J_{a}-R_{a}\right)\frac{\partial H}{\partial x}\left(x\right)+\left(J_{d}-R_{d}\right)\frac{\partial H_{a}}{\partial x}\left(x\right)\right]=0.$$

When the matrix-valued functions *J*, *R*, and *G* in (1) depend on the state variable *x*, it is not straightforward to derive a solution to (25) from its continuous-time counterpart (26). However, in the constant case considered in this paper, the function $H_{a}$ and the matrices $J_{a}$ and $R_{a}$ that satisfy (26) also satisfy (25). This result is summarized in the following corollary.

**Corollary** **1.** *Let us assume that there exists $H_{a}:X\to R$, $J_{a},\;R_{a}\in R^{n\times n}$ and $G^{⊥}\;\in R^{m\times n}$ such that the continuous-time matching Equation (26) holds for the port-Hamiltonian system (1). Then, $H_{a}$, $J_{a}$ and $R_{a}$ are also solutions of the discrete-time reformulation (25).*

**Proof.**  If we consider, for example, the mean value discrete gradient (6), since (26) holds for all x∈X, the result follows by replacing *x* with $\left(1-\sigma \right)x_{k}+\sigma x_{k+1}$, where σ∈R, and then integrating with respect to σ. □

**Remark** **3.** *Since the control action (14) with $\beta (x_{k},x_{k+1})$ defined in (22) and $v_{k}$ in (19) depends on $x_{k+1}$, we have to investigate how to compute $u_{k}$ at step k. The first important assumption is that the closed-loop dynamics obtained from (7), (14) and (19) is well-posed; see [20] (Proposition 2.1). The corresponding state equation is*

(27) $$x_{k+1}=x_{k}+\tau \left[J_{d}-\left(R_{d}+GK_{z}G^{T}\right)\right]\bar{\nabla }H_{d}\left(x_{k},x_{k+1}\right).$$

*For (27) at each step k, we let*

$$F_{d,k}\left(\xi \right):=x_{k}+\tau \left[J_{d}-\left(R_{d}+GK_{z}G^{T}\right)\right]\bar{\nabla }H_{d}\left(x_{k},\xi \right),$$

*where $\xi \in R^{n}$. So, ξ is the solution of $\xi =F_{d,k}\left(\xi \right)$, with $\xi \equiv x_{k+1}$ only in the nominal case, i.e., when the plant exactly behaves as (27), while (14) and (19) provide $u_{k}$. This approach is conceptually similar to [9], where the control design problem is tackled for discrete-time models of mechanical systems in port-Hamiltonian form arising from symplectic integration schemes (i.e., the implicit midpoint rule). On the other hand, it differs from [12,18] where the implicit state equation is approximated with an explicit one.*

**Remark** **4.** *The key step in the procedure of Proposition 1 is to solve (21) for suitable matrices ${\bar{K}}_{a}$, $J_{a}$, and $R_{a}$, where ${\bar{K}}_{a}$ must be the discrete gradient of a function $H_{a}$. If the structure matrices of the port-Hamiltonian system (1) are not constant, Corollary 1 cannot be applied, and solving the matching equation may become considerably more involved. A different approach has been discussed in [20] (Section III.B). The idea is to exploit the algebraic nature of (21) to derive a broader class of control laws of the form (22), capable of performing an (approximate) energy-shaping action on the open-loop system (7) without requiring ${\bar{K}}_{a}$ to be the discrete gradient of an energy function. This approach is the discrete-time extension of the technique proposed in [30]. This topic is beyond the scope of the present paper.*

## 4. “Performance-Preserving” Coupling Strategies

The aim of this section is to present a strategy for coupling the discrete-time regulator designed according to the procedure illustrated in Section 3 with the continuous-time plant (1). Since the controller is designed on the basis of the discrete-time model (7), its performance is typically assessed through numerical simulations using the same model as a reference. The proposed coupling strategy therefore aims at applying the “smallest” perturbation to the nominal stabilizing control law that guarantees performance as close as possible to that of the reference case. In particular, the transient and steady-state behaviors are preserved if the variation in the closed-loop Hamiltonian function $H_{d}$ defined in (17) along the system trajectories remains unchanged. These considerations lead to an optimization problem that must be solved at each step k∈N, resulting in a novel digital-to-analog interface.

For any energy-based control synthesis methodology such as the ones that fit into the framework illustrated in Section 3, let us consider a coupling algorithm based on the solution of the following nonlinear optimization problem at each step k∈N [27]:

(28) $$\begin{array}{cc} \; & \;\min_{u_{\delta },x\left(t\right)}{∥u_{\delta }∥}^{2}\\ s.t.\; & \dot{x}\left(t\right)=\left[J(x(t))-R(x(t))\right]\nabla H\left(x\left(t\right)\right)+G\left(x\left(t\right)\right)\left[\beta \left(x_{k},x_{k+1}\right)+u_{\delta }\right]\\ \; & x\left(t_{k}\right)=x_{k}\\ \; & \beta \left(x_{k},x_{k+1}\right)+u_{\delta }\in U\\ \; & {∥u_{\delta }∥}^{2}\leq \Delta \\ \; & H_{d}\left(x\left(t_{k+1}\right)\right)\leq H_{d}\left(x_{k+1}\right). \end{array}$$

The concept is straightforward. At each $t_{k}$, the state of (1) is measured, and the control action in (14) is determined using the discrete-time model (7), with $x_{k}=x\left(t_{k}\right)$. For simplicity, and without loss of generality, we assume $v_{k}=0$ in (14); hence, the required dissipation is provided by the state-feedback law β. As explained in Remark 3, the design procedure yields the “next” state $x_{k+1}$ together with $u_{k}=\beta \left(x_{k},x_{k+1}\right)$. Therefore, in the ideal setting, the closed-loop energy at $t_{k+1}$ is known and is equal to $H_{d}\left(x_{k+1}\right)$. The digital-to-analog interface must determine the “smallest” perturbation $u_{\delta }\in R^{m}$ of the nominal input $\beta (x_{k},x_{k+1})$ such that the control action $u\left(t\right)=\beta \left(x_{k},x_{k+1}\right)+u_{\delta }$, applied to (1) over $\left[t_{k},t_{k+1}\right)$, is admissible, i.e., belongs to *U*, and ensures that $H_{d}$ evaluated at $x(t_{k+1})$ does not exceed $H_{d}\left(x_{k+1}\right)$. As a result, the closed-loop energy function decreases at least as rapidly as in the reference discrete-time system. The parameter Δ>0 specifies an upper bound on $u_{\delta }$.

**Remark** **5.** *Feasibility of (28) cannot always be ensured. This is the main limitation of the proposed approach, since a perturbation $u_{\delta }$ must be computed at every $t_{k}$ to obtain the control action $u_{k}$. As in [31] (Theorem 2.5), the Gronwall–Bellman inequality can be used to show that, at some sampling instants, $H_{d}$ may fail to decrease as intended; namely, $x(t_{k+1})$ may not lie in the “desired” level set defined by the final inequality constraint in (28). A related issue was addressed in [25] by dissipating, over the subsequent sampling interval(s), the difference between the achieved and desired energy at $t_{k+1}$. Although this strategy can also be adopted here, it is outside the scope of this paper. Soft constraints provide a simple means of addressing the feasibility issue in (28). The resulting optimization problem is*

(29) $$\begin{array}{cc} \; & \;\min_{u_{\delta },\varepsilon ,x\left(t\right)}{∥u_{\delta }∥}^{2}+K_{\varepsilon }\varepsilon^{2}\\ s.t.\; & \dot{x}\left(t\right)=\left[J(x(t))-R(x(t))\right]\nabla H\left(x\left(t\right)\right)+G\left(x\left(t\right)\right)\left[\beta \left(x_{k},x_{k+1}\right)+u_{\delta }\right]\\ & x\left(t_{k}\right)=x_{k}\\ & H_{d}\left(x\left(t_{k+1}\right)\right)\leq H_{d}\left(x_{k+1}\right)+\varepsilon^{2}. \end{array}$$

*Here, the amplitude constraint on $u_{\delta }$ has been removed and $U\equiv R^{m}$ is assumed. In (29), choosing $K_{\varepsilon }\gg 1$ allows the variable ε∈R to relax dynamically the constraint on the closed-loop energy function $H_{d}$ at $t_{k+1}$. The objective is to determine the “smallest” correction $u_{\delta }$ to the nominal control action $\beta (x_{k},x_{k+1})$ such that $H_{d}\left(x\left(t_{k+1}\right)\right)$ is as close as possible to the target value $H_{d}\left(x_{k+1}\right)$. Selecting a sufficiently large $K_{\varepsilon }$ reduces this mismatch. It is straightforward to see that (29) is always feasible and that a solution exists because the cost function is lower-bounded.*

Noticeable limitations of (28) and (29) are that the constraint on x(t) depends on a nonlinear ODE and that the closed-loop Hamiltonian function $H_{d}\left(x\right)$ is, in general, non-quadratic. Consequently, the real-time implementation of the coupling mechanism is potentially computationally demanding. Under a few conditions, an explicit solution is possible when the plant is linear and the closed-loop Hamiltonian is quadratic. The result is based on [32] (Proposition 1), as reported below.

**Proposition** **2.** *Let $a\in R^{\bar{n}}$, $P\in R^{\bar{n}\times \bar{n}}$ and such that $P=P^{T}>0$, and r∈R, with r>0. The solution $v_{★}$ of the convex optimization problem*

(30) $$\begin{array}{cc} \; & \;\min_{v}{\left(v-a\right)}^{T}P\left(v-a\right)\\ s.t.\; & {\left(v-c\right)}^{T}P\left(v-c\right)\leq r^{2} \end{array}$$

*is*

(31) $$v_{★}=\left\{\begin{array}{cc} a & \mathrm{if}\;{\left(a-c\right)}^{T}P\left(a-c\right)\leq r^{2}\\ \frac{r(a-c)}{\sqrt{{\left(a-c\right)}^{T}P\left(a-c\right)}}+c & otherwise. \end{array}\right.$$

Let us consider the linear port-Hamiltonian system (9), which can be rewritten in the (A,B,C) form with A=(J−R)Q, B=G, and $C=G^{T}Q$. Let us also assume that the discrete-time control design procedure assigns target dynamics in port-Hamiltonian form with quadratic Hamiltonian

(32) $$H_{d}\left(x\right)=\frac{1}{2}{\left(x-x_{★}\right)}^{T}Q_{d}\left(x-x_{★}\right),\;Q_{d}=Q_{d}^{T}>0.$$

With simple computations, it is easy to see that, at the end of the *k*-th sampling interval, starting from $x\left(t_{k}\right)=x_{k}$ and under the control input $u_{k}=\beta \left(x_{k},x_{k+1}\right)+u_{\delta }$, (9) reaches

(33) $$x\left(t_{k+1}\right)=A_{k}+B_{\delta }u_{\delta },$$

where

$$\begin{array}{cc} A_{k} & =e^{A\tau }\left[x_{k}+\left(\int_{0}^{\tau }e^{-A\sigma }\;d\sigma \right)B\beta \left(x_{k},x_{k+1}\right)\right]\\ B_{\delta } & =e^{A\tau }\left(\int_{0}^{\tau }e^{-A\sigma }\;d\sigma \right)B. \end{array}$$

Consequently, the energy constraint $H_{d}\left(x\left(t_{k+1}\right)\right)\leq H_{d}\left(x_{k+1}\right)$ in (28) takes the form of the quadratic constraint in (30), with $P=B_{\delta }^{T}Q_{d}B_{\delta }$, $c=B_{\delta }^{+}\left(x_{★}-A_{k}\right)$, and $r^{2}=2H_{d}\left(x_{k+1}\right)$. Thus, the coupling strategy is recast as the optimization problem of Proposition 2, in which a=0. Consequently,

$$u_{\delta }=\left\{\begin{array}{cc} 0 & \mathrm{if}\;\frac{1}{2}{∥x_{★}-A_{k}∥}_{Q_{d}}^{2}\leq H_{d}\left(x_{k+1}\right)\\ \left[1-\frac{\sqrt{2H_{d}\left(x_{k+1}\right)}}{{∥x_{★}-A_{k}∥}_{Q_{d}}}\right]\left(x_{★}-A_{k}\right) & \mathrm{otherwise}, \end{array}\right.$$

where ${∥x_{★}-A_{k}∥}_{Q_{d}}^{2}={\left(x_{★}-A_{k}\right)}^{T}Q_{d}\left(x_{★}-A_{k}\right)$. Note that this result can be used to address the general nonlinear case approximately, by relying, at each sampling interval k∈N, on a linear approximation of the plant dynamics around $x_{k}$.

## 5. Numerical Example: The 2-Dof Planar Robotic Manipulator

**Figure 1:**
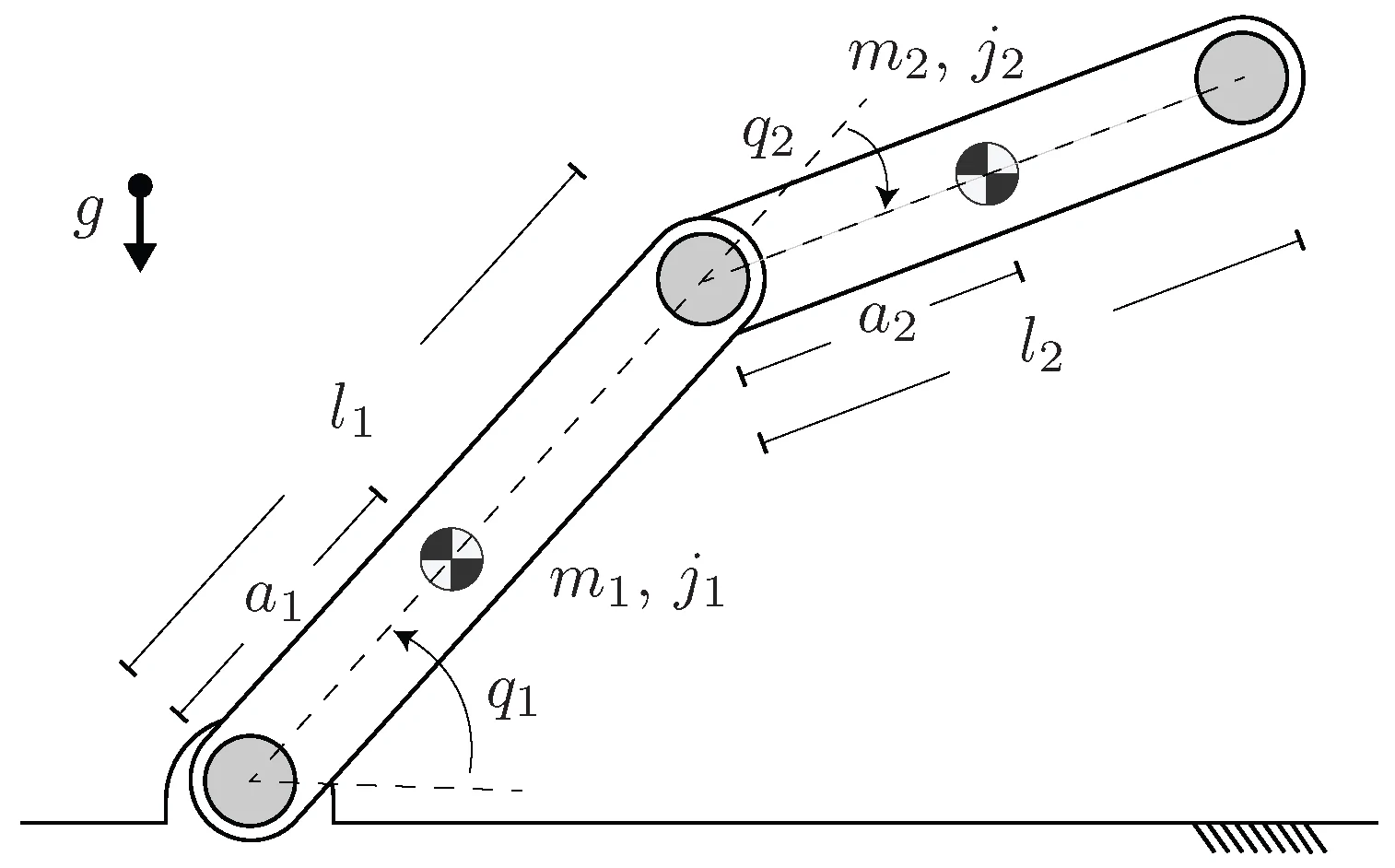
The two degrees-of-freedom planar manipulator [27].

As in [27], let us consider the two degrees-of-freedom planar manipulator of Figure 1, which belongs to the class illustrated in Example 2.

The generalized coordinates are $q=\left(q_{1},q_{2}\right)\in R^{2}$, and the total energy is (12) in which the inertia matrix is

$$M\left(q\right)=\left(\begin{array}{cc} j_{1}+j_{2}+m_{1}a_{1}^{2}+m_{2}\left(l_{2}^{2}+a_{2}^{2}+2a_{2}l_{1}\cos q_{2}\right) & j_{2}+m_{2}\left(a_{2}^{2}+a_{2}l_{1}\cos q_{2}\right)\\ j_{2}+m_{2}\left(a_{2}^{2}+a_{2}l_{1}\cos q_{2}\right) & j_{2}+m_{2}a_{2}^{2} \end{array}\right)$$

and the potential energy is

(34) $$V\left(q\right)=m_{1}ga_{1}\sin q_{1}+m_{2}g\left[l_{1}\sin q_{1}+a_{2}\sin\left(q_{1}+q_{2}\right)\right],$$

with *g* being the gravity acceleration. The continuous-time model is (11), with B(q)=I and D=dI, where d≥0. The inputs $u\left(t\right)\in R^{2}$ are the torques at each joint. The parameters are reported in Table 1.

The discrete-time approximation of the plant model is (13), and in the numerical examples, the mean value discrete gradient (6) is employed. Note that from (34), for any $q,\;q_{+}\in R^{2}$, we have that

(35) $$\bar{\nabla }V\left(q,q_{+}\right)=\left(\begin{array}{c} {\bar{\nabla }}_{q_{1}}V\left(q,q_{+}\right)\\ {\bar{\nabla }}_{q_{2}}V\left(q,q_{+}\right) \end{array}\right),$$

where $q_{+}=\left(q_{1+},q_{2+}\right)$ and

$$\begin{array}{cc} {\bar{\nabla }}_{q_{1}}V\left(q,q_{+}\right) & =\left(m_{1}ga_{1}+m_{2}gl_{1}\right)\frac{\sin q_{1+}-\sin q_{1}}{q_{1+}-q_{1}}+m_{2}ga_{2}\frac{\sin\left(q_{1+}+q_{2+}\right)-\sin\left(q_{1}+q_{2}\right)}{q_{1+}+q_{2+}-q_{1}-q_{2}}\\ {\bar{\nabla }}_{q_{2}}V\left(q,q_{+}\right) & =m_{2}ga_{2}\frac{\sin\left(q_{1+}+q_{2+}\right)-\sin\left(q_{1}+q_{2}\right)}{q_{1+}+q_{2+}-q_{1}-q_{2}}. \end{array}$$

Based on the general framework summarized in Proposition 1, it is easy to check that the matching Equation (21) with $J_{a}=R_{a}=0$ is satisfied if $H_{a}$ depends on the displacements $q_{k}$ only. As a consequence, the control action β only shapes the potential energy. The simplest choice is to have $H_{a}\left(q\right)=-V\left(q\right)+\frac{1}{2}{\left(q-q_{★}\right)}^{T}K_{p}\left(q-q_{★}\right)$, with $K_{p}=K_{p}^{T}>0$, so that the desired Hamiltonian function $H_{d}\left(q,p\right)=H\left(q,p\right)+H_{a}\left(q\right)$ has a global minimum in $x_{★}$, which means that the conditions (23) are satisfied. It is interesting to note that an explicit expression for β can be computed based on (35) and on the fact that the further term in $H_{a}$ is quadratic. This leads to the PD plus gravity compensation controller. Asymptotic convergence to the global minimum $x_{★}$ of $H_{d}\left(q,p\right)$ is obtained via the damping injection law (19), where $z_{k}=y_{k}={\bar{\nabla }}_{p}H\left(x_{k},x_{k+1}\right)$ is the output of either the plant and of the closed-loop system. As far as the controller parameters are concerned, we have $K_{p}=60\;I$ and $K_{z}=20\;I$.

**Figure 2:**
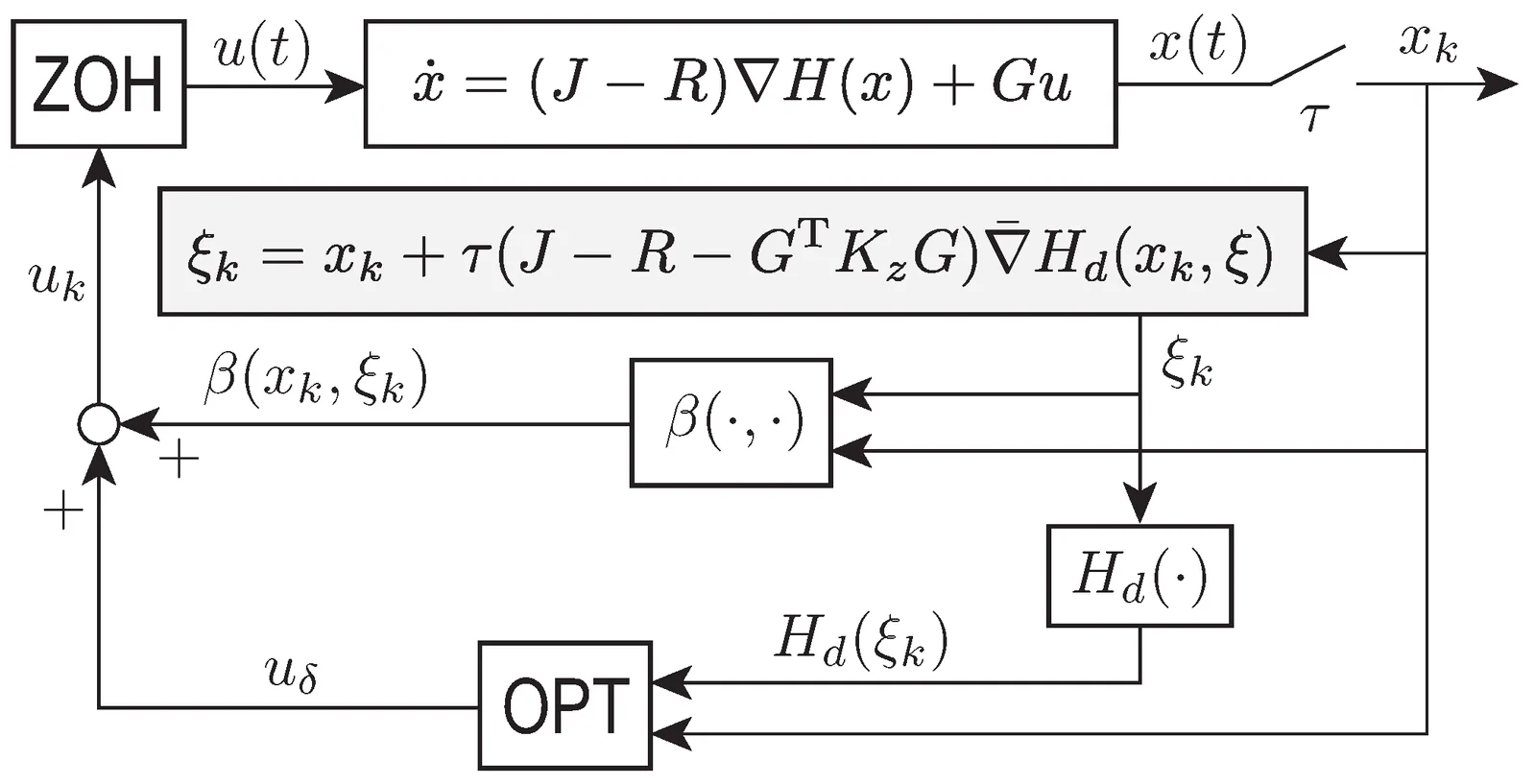
Schematic representation of the closed-loop system [27].

The block diagram of the overall system, obtained by coupling the discrete-time controller with the continuous-time plant, is shown in Figure 2. It illustrates the practical implementation of the control action.

Since the stabilizing law relies on state feedback, the procedure starts by sampling the plant state x(t) at $t=t_{k}$. The resulting variable $x_{k}$ is then used to compute the “desired” next state $\xi_{k}$ according to the target dynamics. Such dynamics belong again to the class illustrated in Example 2, in which $D=d\;I+K_{z}$, $u_{k}=0$ and *H* is replaced by $H_{d}\left(q,p\right)=\frac{1}{2}p^{T}M^{-1}\left(q\right)p+\frac{1}{2}q^{T}K_{p}q$. As explained in Remark 3, the discrete-time variable $\xi_{k}$ is introduced to distinguish it from $x_{k+1}$, which denotes the value of x(t) sampled at $t=t_{k+1}$. Computing $\xi_{k}$ requires solving an implicit equation, represented by the gray block. The control action β is then determined from $x_{k}=\left(q_{k},p_{k}\right)$ and $\xi_{k}=\left({\hat{q}}_{k},{\hat{p}}_{k}\right)$ and takes the following form:

$$\beta \left(x_{k},\xi_{k}\right)=-\bar{\nabla }V\left(q_{k},{\hat{q}}_{k}\right)+\frac{1}{2}K_{p}\left(q_{k}+{\hat{q}}_{k}\right)-K_{z}{\bar{\nabla }}_{p}H_{d}\left(x_{k},\xi_{k}\right).$$

**Figure 3:**
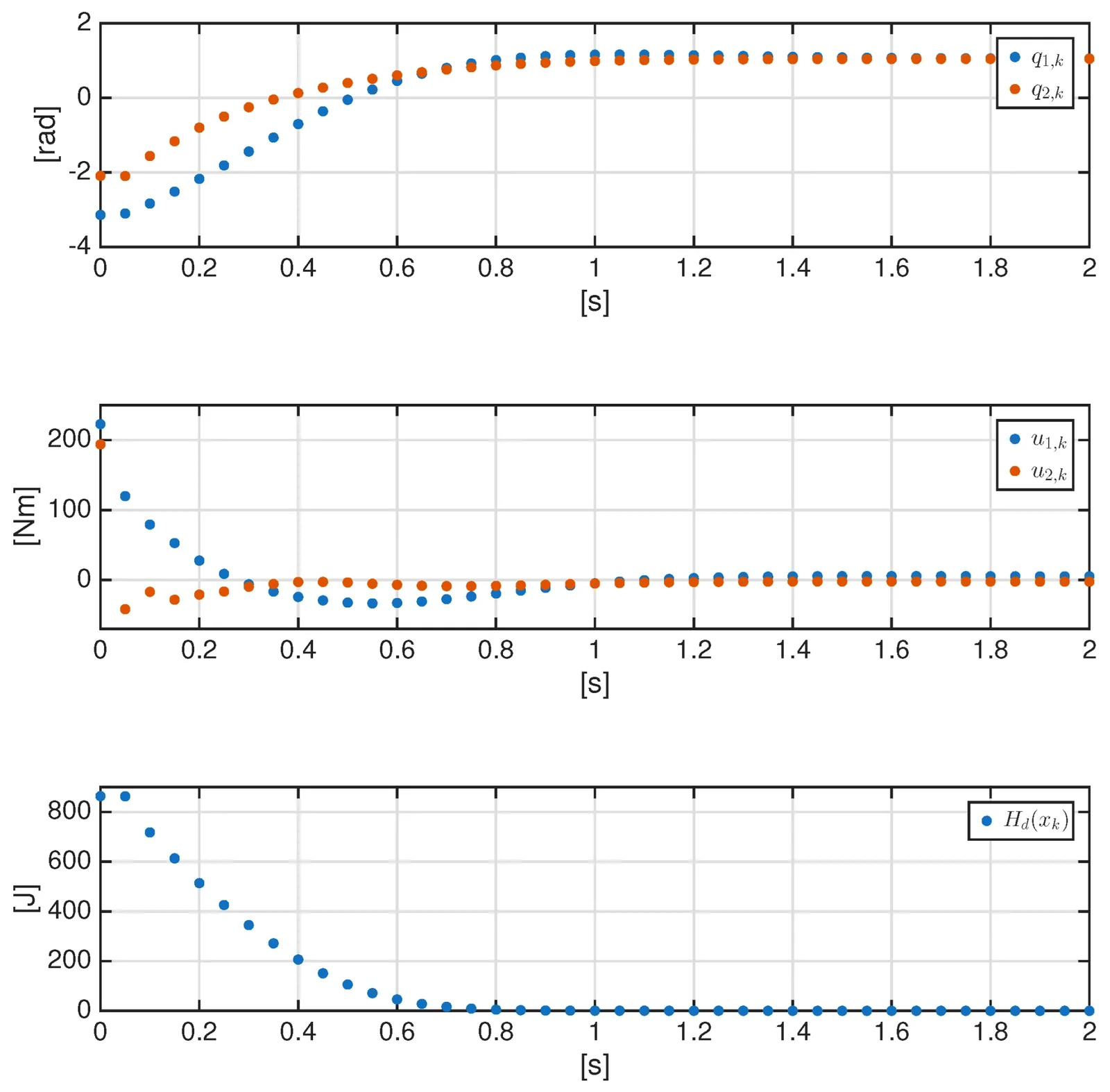
Response in discrete-time (reference scenario) [27].

However, whereas the “corrective” term $u_{\delta }$ is obtained using the strategies described in Section 4. In both cases, we set $x_{k+1}\equiv \xi_{k}$. We first consider the discrete-time behavior, namely the response obtained when the control law is applied to the discrete-time plant model (13), shown in Figure 3.

This response represents the desired behavior that the designer seeks to reproduce when the digital controller is interconnected with the “real” plant, or equivalently, in this case, with its continuous-time model (11). The control law is synthesized and the regulator parameters are tuned in the discrete-time framework, with the aim of achieving comparable performance in the real-world implementation. Starting from the upper plot in Figure 3, the evolution of the discrete displacements $q_{k}$, the control input $u_{k}$, and the closed-loop Hamiltonian function $H_{d}\left(x_{k}\right)$ is reported. The latter captures the transient behavior of the controlled system and provides the key information required by the coupling techniques presented in Section 4.

**Figure 4:**
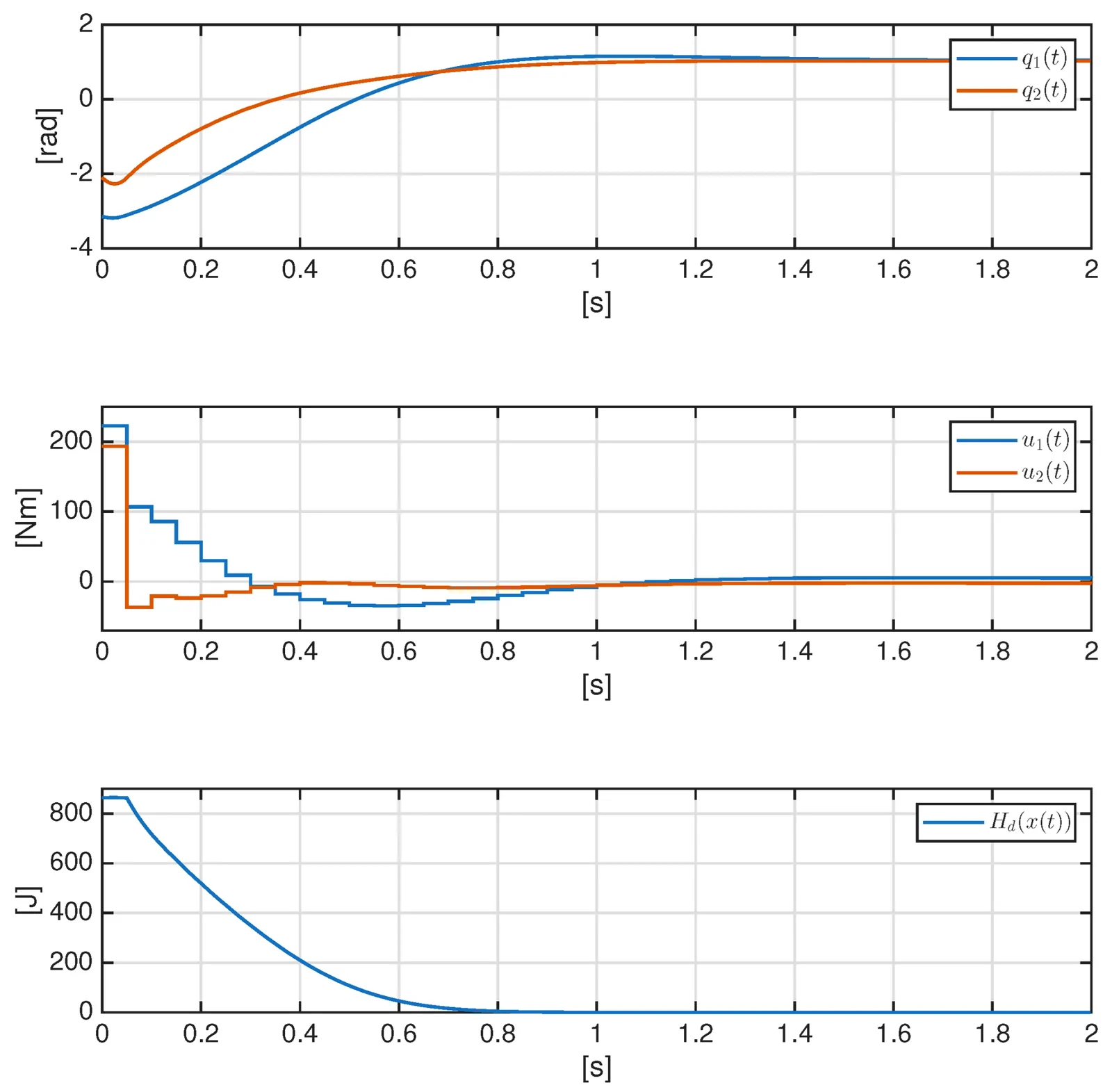
Response with $u_{\delta }$ obtained thanks to (29) [27].

**Figure 5:**
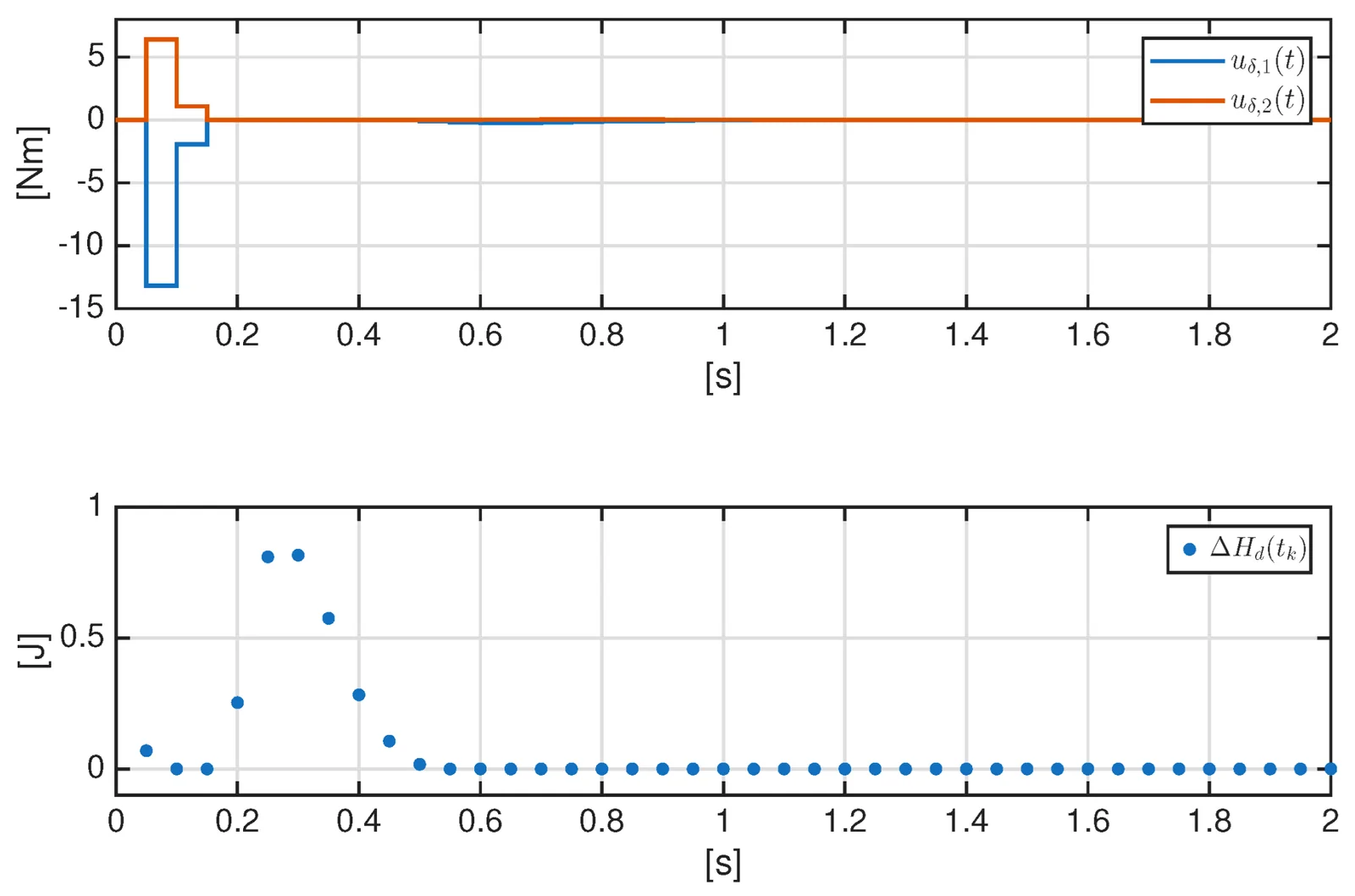
Behaviour of the coupling strategy based on (29) [27].

We next examine the coupling strategy based on the optimization problem (29), with $K_{\varepsilon }=10^{4}$. This sub-optimal version of (28) is adopted because it does not present feasibility issues. Nevertheless, for the particular example considered here, (28) is always feasible. The closed-loop behavior is shown in Figure 4 and Figure 5. Comparing Figure 3 with Figure 4 reveals that coupling the discrete-time controller with the continuous-time plant yields performance that is practically identical to that of the reference case. The role of the interconnection mechanism is illustrated in Figure 5. The corrective term $u_{\delta }$ differs from 0 only during the initial transient, and its magnitude is considerably smaller than that of the nominal control action β. In this specific case, the discrete-time design procedure based on the class of implicit port-Hamiltonian systems (7) or (13) already provides very good performance. Therefore, from a “practical” real-world perspective, the constraint on $∥u_{\delta }∥$, namely the parameter Δ in the optimization problem (28), is not strictly required. This observation also motivates the formulation of the optimization problem (29), whose purpose is to avoid feasibility issues. The lower plot in Figure 5 reports the evolution of $\Delta H_{d}\left(t_{k}\right)=H_{d}\left(\xi_{k-1}\right)-H_{d}\left(x\left(t_{k}\right)\right)$. This quantity measures the difference between the target and the achieved closed-loop Hamiltonian at each sampling instant. More specifically, for every sampling interval *k*, the variable $\xi_{k}$ is computed as the next state associated with the desired discrete-time evolution of the controlled system. Consequently, $H_{d}\left(\xi_{k}\right)$ is the target closed-loop energy value at the end of the sampling interval, namely at $t_{k+1}$. The coupling strategy is required to ensure that $H_{d}\left(x\left(t_{k+1}\right)\right)$ is lower than or equal to $H_{d}\left(\xi_{k}\right)$. The behavior of $\Delta H_{d}\left(t_{k}\right)$ in Figure 5 is consistent with this requirement, confirming that the reference performance of the discrete-time setting is recovered in the mixed discrete-continuous-time case.

## 6. Conclusions and Future Activities

This paper presented a complete synthesis procedure for the design and implementation of energy-based digital controllers for port-Hamiltonian systems. The procedure consists of three main steps. First, a discrete-time port-Hamiltonian approximation of the plant is obtained by “replacing” the Hamiltonian gradient with a discrete gradient. This construction preserves the energy balance relation of the continuous-time system, although it generally leads to implicit state equations. Second, an extension of the IDA-PBC methodology to the resulting class of discrete-time systems is presented. The proposed design reshapes the Hamiltonian function and modifies the interconnection and dissipative structures in order to assign a desired equilibrium and guarantee its asymptotic stability under suitable conditions. The third step addresses the interconnection between the digital controller and the continuous-time plant. A performance-preserving digital-to-analog interface is proposed, based on the solution of an optimization problem that computes the smallest correction to the nominal control action required to enforce a desired decrease in the closed-loop Hamiltonian over each sampling interval. A soft-constrained formulation is also considered to address possible feasibility issues. For linear systems with quadratic desired Hamiltonians, the optimization problem admits an explicit solution. The complete procedure was illustrated using a two-degree-of-freedom planar manipulator. The numerical results show that the proposed interface allows the mixed discrete-continuous-time closed-loop system to reproduce closely the response obtained in the reference discrete-time design scenario, while requiring only a small correction to the nominal control action, mainly during the initial transient.

Future work will focus on extending the proposed discrete-time IDA-PBC framework to systems with state-dependent structure matrices, for which solving the matching equation is more challenging. Further research will also address computationally efficient implementations of the coupling strategy for nonlinear systems, the treatment of feasibility issues over successive sampling intervals, and experimental validation on physical systems.

## Figures and Tables

**Table 1:** Parameters of the robot in Figure 1; all quantities are expressed in SI units [27].

Parameter	Value	Parameter	Value
$m_{1}=m_{2}$	1	$j_{1}=j_{2}$	0.1
$l_{1}=l_{2}$	1	$a_{1}=a_{2}$	0.5
*d*	0.01	τ	0.05
$\left(q_{0},p_{0}\right)$	$-\left(\pi ,\frac{2}{3}\pi ,5,5\right)$	$q_{★}$	$\left(\frac{\pi }{3},\frac{\pi }{3}\right)$

## Data Availability

The original contributions presented in this study are included in the article. Further inquiries can be directed to the corresponding author.
